# Combination of Gefitinib and DNA Methylation Inhibitor Decitabine Exerts Synergistic Anti-Cancer Activity in Colon Cancer Cells

**DOI:** 10.1371/journal.pone.0097719

**Published:** 2014-05-29

**Authors:** Yun-feng Lou, Zheng-zhi Zou, Pin-jia Chen, Guo-bin Huang, Bin Li, De-qing Zheng, Xiu-rong Yu, Xiao-yong Luo

**Affiliations:** 1 Department of Oncology, The Affiliated Luoyang Central Hospital of Zhengzhou University, Luoyang, China; 2 MOE Key Laboratory of Laser Life Science and Institute of Laser Life Science, College of Biophotonics, South China Normal University, Guangzhou, China; 3 Department of Gastroenterology, The Affiliated Donghua Hospital of Sun Yat-sen University, Dongguan, China; Southern Illinois University School of Medicine, United States of America

## Abstract

Despite recent advances in the treatment of human colon cancer, the chemotherapy efficacy against colon cancer is still unsatisfactory. In the present study, effects of concomitant inhibition of the epidermal growth factor receptor (EGFR) and DNA methyltransferase were examined in human colon cancer cells. We demonstrated that decitabine (a DNA methyltransferase inhibitor) synergized with gefitinib (an EGFR inhibitor) to reduce cell viability and colony formation in SW1116 and LOVO cells. However, the combination of the two compounds displayed minimal toxicity to NCM460 cells, a normal human colon mucosal epithelial cell line. The combination was also more effective at inhibiting the AKT/mTOR/S6 kinase pathway. In addition, the combination of decitabine with gefitinib markedly inhibited colon cancer cell migration. Furthermore, gefitinib synergistically enhanced decitabine-induced cytotoxicity was primarily due to apoptosis as shown by Annexin V labeling that was attenuated by z-VAD-fmk, a pan caspase inhibitor. Concomitantly, cell apoptosis resulting from the co-treatment of gefitinib and decitabine was accompanied by induction of BAX, cleaved caspase 3 and cleaved PARP, along with reduction of Bcl-2 compared to treatment with either drug alone. Interestingly, combined treatment with these two drugs increased the expression of XIAP-associated factor 1 (XAF1) which play an important role in cell apoptosis. Moreover, small interfering RNA (siRNA) depletion of XAF1 significantly attenuated colon cancer cells apoptosis induced by the combination of the two drugs. Our findings suggested that gefitinib in combination with decitabine exerted enhanced cell apoptosis in colon cancer cells were involved in mitochondrial-mediated pathway and induction of XAF1 expression. In conclusion, based on the observations from our study, we suggested that the combined administration of these two drugs might be considered as a novel therapeutic regimen for treating colon cancer.

## Introduction

Colon cancer is one of the most commonly occurring tumors and a major cause of cancer-related deaths worldwide, which accentuates the need for effective strategies to prevent and treat this malignancy. Therapies available for treatment of colon cancer include surgery, radiation therapy, chemotherapy, immunomodulatory therapy, and molecularly targeted therapies such as anti-vascular endothelial growth factor receptor (VEGFR) and anti-epidermal growth factor receptor (EGFR) therapy [1], [2]. Among these molecularly targeted treatments, EGFR-targeted therapy as one of the important clinical strategies has been increasingly widely applied in patients with metastatic colon cancer [3].

EGFR is a transmembrane glycoprotein with an extracellular EGF-binding domain and an intracellular region containing the tyrosine kinase domain that regulates signaling pathways to control cell proliferation, differentiation, drug and radiation sensitivity, and angiogenesis [4]. Expression of a high level of EGFR has been found in various types of human cancers, including colon cancer, gastric cancer, lung cancer, breast cancer, and squamous cell carcinoma of head and neck [5], [6], [7], [8], [9], [10]. Overexpression and constitutive activation of EGFR have been associated with a poor prognosis in colon cancer patients. As activation of EGFR is correlated with colon cancer progression, the EGFR has been the target of anticancer drug development efforts. These strategies targeting EGFR include using monoclonal antibodies such as cetuximab, and small molecular tyrosine kinase inhibitors (TKI) such as gefitinib and erlotinib. Gefitinib is a synthetic anilinoquinazoline and orally active selective EGFR-TKI that blocks the signal transduction pathway involved in the proliferation and survival of cancer cells. In a clinical setting, gefitinib treatment has been approved for various types of cancer including colon cancer [11]. However, the emerging clinical experience has disappointingly revealed that despite gefitinib demonstrating some antitumor activity in colon cancer, there is a high level of *de novo* resistance to such treatment [12]. Hence, efforts are ongoing for the development of anti-colon cancer regimens that would combine gefitinib with other drugs.

Epigenetic modifications, mainly DNA methylation and histones acetylation, are now recognized as the main mechanisms contributing to tumor malignant phenotypes [13]. As a consequence, several drugs that affect epigenetic pathways have been approved for cancer treatment and more are currently in clinical trials [14], [15]. Notably, the reversibility of epigenetic modifications by small-molecule inhibitors means that off target effects should be minimal and reversible upon cessation of treatment. Recently, DNA methyltransferase inhibitors are at a more clinically advanced stage of development than the inhibitors of histone deacetylases or histone methyltransferases, having been extensively tested in phase I–III clinical trials [16]. The archetypal DNA methyltransferase inhibitor decitabine (i.e., 5-aza-2′-deoxycytidine), a deoxyribose analogue of 5-azacytidine, is currently used to treat myelodysplastic syndrome (MDS), and is under investigation for treating acute myeloid leukemia (AML) and other solid cancer [17], [18]. Moreover, decitabine and suberanilohydroxamic acid (SAHA, a histone deacetylase inhibitor) cooperate to sensitize colon cancer cells to Fas ligand-induced apoptosis [19].

Currently, major efforts have been made to develop some anticancer therapies based on the small molecules that specifically target DNA demethylating protein or EGFR, whereas, not much information is known about the combined effects of EGFR inhibitors and demethylating agents in colon cancer. Previous studies showed gefitinib could abate chemotherapy resistance by inhibiting transmembrane transporters of the ABC family, including the P-glycoprotein (P-gp), the multidrug resistance protein 1 (MRP1) and the breast cancer resistance protein (BCRP) [20]. Based on these premises, we decided to determine if the combination of gefitinib and decitabine has synergistic activity in colon cancer cells.

In the current study, we provided preclinical data that showed the combination of gefitinib and decitabine was synergistic at inhibiting cell proliferation, migration and inducing apoptosis in cultured human colon cancer cells. Furthermore, we provided the evidences that gefitinib combined with decitabine regulated cell apoptosis were involved in mitochondrial-mediated pathway and induction of XAF1 expression. Taken together, these accumulating data may guide development of new colon cancer therapies.

## Materials and Methods

### Cell Culture, Reagents and Drugs Treatment

The colon cancer-derived cell lines SW1116 and LOVO were obtained from the American Type Culture Collection (ATCC) and grown in DMEM medium (Gibco; Life Technologies, Carlsbad, CA) supplemented with 10% (v/v) fetal bovine serum (FBS) (Gibco; Life Technologies, Carlsbad, CA) at 37°C in 5% CO_2_ incubator. Cells were grown in monolayer and passaged routinely 2–3 times a week. decitabine and gefitinib were purchased from Selleck Chemicals LLC (Houston TX, USA). MTT [3-(4,5-Dimethylthiazol-2-yl)-2,5-diphenyltetrazolium bromide], dimethyl sulfoxide (DMSO), z-Val-Ala-Asp-fluoromethylketone (z-VAD-fmk), necrostatin-1, necrostatin-5 were purchased from Sigma (St. Louis, MO, USA). For drugs treatment, decitabine, gefitinib, z-VAD-fmk, necrostatin-1, and necrostatin-5 were dissolved in DMSO respectively, aliquots were stored at −80°C. Stock solutions were diluted to the desired final concentrations with growth medium just before use. Prior to drugs treatment, cells were incubated for at least 12 h and thereafter replaced with media containing drugs; DMSO-treated cells were used as a mock control.

### Cell Viability, Clonogenic Cell Survival and Apoptosis Assays

Cell viability was assessed using standard MTT assay. Briefly, cells were plated at a density of 0.5–1×10^4^ cells per well in 96-well plates and incubated for 12 h in a 5% CO_2_ atmosphere at 37°C before treatment of exposed to drugs. The media were then removed, and the cells were treated with decitabine and/or gefitinib. After the cells were incubated for 48 h, 100 µL MTT solutions (2 mg/mL) were added to each well and the plate was incubated for another 4 h at 37°C. The formed formazan crystals were dissolved in DMSO (200 µL per well) with constant shaking for 5 min. Absorbance of the solution was then measured using a Micro-plate Reader (Bio-Rad, Hercules, CA) at 495 nm. This assay was conducted in triplicate.

For clonogenic cell survival experiments, the attached cells from the same 10 cm culture dish that were trypsinized with 1 mL trypsin–EDTA (Gibco; Life Technologies, Carlsbad, CA) and inactivated with media containing 10% FBS. The cells were counted using a hemocytometer and plated at low density (1000 per well in six well plate). After 24 h, drugs were added at indicated concentration for 24 h. After drugs removal, cells were allowed to proliferate in a humidified 5% CO_2_, 37°C environment for 15 days in fresh medium. Cells were fixed in 70% ethanol and stained with 0.005% crystal violet (sigma, St. Louis, MO, USA) for analysis of clonogenic cell survival as previously described [21]. The colony forming units with more than 100 cells were counted using a light microscope.

Measurement of apoptosis was conducted by Annexin V-FITC (fluorescein isothiocyanate)/PI (propidium iodide) analysis as described previously [22]. Briefly, cells were seeded and treated with the drugs for 48 h. Afterward, the cells were washed twice with PBS and 1×10^6^ cells were resuspended in 1 mL of 1× Annexin V binding buffer. Cells undergoing apoptotic cell death were analyzed by counting the cells that stained positive for Annexin V-FITC and negative for PI, and late stage of apoptosis as Annexin V-FITC and PI positive using FACS Calibur flow cytometer (BD Biosciences, San Jose, CA, USA).

### Combination Index

For combination treatment of decitabine and gefitinib, MTT assay data were converted to fraction of growth affected by the individual drug or the combination treated cells compared with untreated cells and analysed using CalcuSyn software (Biosoft, Ferguson, MO, USA) to determine whether the combination was synergistic. This program is based upon the Chou–Talalay equation [23], which calculates a combination index (CI). The general equation for the classic isobologram is given by: CI = (D)_1_/(Dx)_1_+(D)_2_/(Dx)_2_. Where Dx indicates the dose of one compound alone required to produce an effect, (D)_1_ and (D)_2_ are the doses of compounds 1 and 2, respectively, necessary to produce the same effect in combination. From this analysis, the combined effects of the two compounds can be summarized as follows: CI<1, CI = 1, CI>1 indicate synergistic, additive and antagonistic effects, respectively.

### Western Blot Analysis

Cells were lysed on ice for 30 min in lysis buffer (50 mM Tris-HCl, 150 mM NaCl, 1 mM EDTA, 0.1% SDS, 0.5% deoxycholic acid, 0.02% sodium azide, 1% NP-40, 2.0 mg/mL aprotinin, 1 mM phenylmethylsulfonylfluoride). The lysates were centrifuged at 12,000 rpm for 30 min at 4°C. The protein concentration was determined by Bradford dye method. Equal amounts (30 to 60 µg) of cell extract were subjected to electrophoresis in 6–12.5% sodium dodecyl sulfate-polyacrylamide (SDS-PAGE) and transferred to PVDF membranes (Millipore, Darmstadt, Germany) for antibody blotting. The membranes were blocked and then incubated with p-mTOR (Ser2448), mTOR, AKT1, p-AKT (Ser473), p-S6K, S6K, BAX, Bcl-2, cleaved caspase 3 (Asp175), cleaved PARP (Asp214), and Actin antibodies (all from Cell Signaling Technologies, Massachusetts, USA). And the XAF1, XIAP antibodies were purchased from Abcam (Cambridge, U.K). Subsequently, the membranes were incubated with a HRP-conjugated secondary antibody (Protein Tech Group, Chicago, IL) at room temperature for 1 h. Detection was performed with the ECL kit (GE Healthcare; Munich, Germany), according to the manufacturer's instructions.

### Caspase Activity Assay

Fluorometric assays of caspase activity were carried out by using the substrate Ac-DEVD-AMC (BD Pharmingen, San Diego, CA) for caspase 3 and Ac-IETD-AMC (BD Pharmingen, San Diego, CA) for caspase 8. Briefly, cells were lysed in lysis buffer (10 mM HEPES, 142 mM KCl, 5 mM MgCl2, 1 mM EDTA, 0.2% NP-40 and pH 7.2) with 10 mM DTT. Following incubation for 30 min on ice, samples were centrifuged at 12,000 rpm for 30 min at 4°C and the protein content in supernatants was determined by Bradford dye method. Aliquots of 10 mg/100 mL assay volume were incubated with 140 mM site-specific tetrapeptide substrates Ac-DEVD-AMC for caspase 3 and Ac-IETD-AMC for caspase 8 in a caspase assay buffer (20 mM HEPES, 100 mM NaCl, 1 mM EDTA, 0.01% (w/v) CHAPS, 10% (w/v) sucrose and pH 7.2) with 10 mM DTT for 30 min. The release of the fluorogenic group AMC was determined at 37°C in a VersaFluor Fluorometer (Bio-Rad, Hercules, CA) with excitation at 380 nm and emission at 440 nm.

### RNA Interference

Small interfering RNA (siRNA) for down-regulating XAF1 gene expression was done by transfection of RNA oligonucleotides with lipofectamine 2000 (Invitrogen, USA) according to the manufacturer's instructions. One day before transfection, SW1116 and LOVO cells were plated on a 35-mm culture dish in RPMI-1640 complete medium. After cells reached 50%–60% confluence, they were transfected with siRNAs as previously described [24]. Briefly, cells were placed in 1 mL of siRNA mixture with 100 nM siRNA and 5 µL lipofectamine 2000. After 8 h of transfection, 1 mL of RPMI-1640 complete medium was added, and experiments were conducted 48 h after transfection. Protein levels were analyzed by Western blot. The negative control (NC) siRNA and siRNA against XAF1 were synthesized by Shanghai GenePharma Co. For XAF1: 5′- AUGUUGUCCAGACUCAGAG-3′ [25];

### Extraction of Total RNA and Real-time Quantitative Reverse Transcription PCR

Total RNA was extracted with TRIzol Reagent (Invitrogen, Carlsbad, CA, USA) according to the manufacturer's instructions. cDNA was prepared from total RNA using random primers (Promega, Madison, USA) and the Omniscript RT kit (Qiagen GmbH, Hilden, Germany). The relative levels of mRNA were determined by real-time quantitative reverse transcription-PCR (RT-PCR) using an Eppendorf Realplex Mastercycler (Eppendorf, Hamburg, Germany) and Quantitect SYBR Green PCR kit (Qiagen GmbH, Hilden, Germany). XAF1 Primer sequences used were as follows: 5′-ATGGAAGGAGACTTCTCGGT-3′ and 5′-TTGCTGAGCTGCATGTCCAG-3′ [26]. Actin (BioVision, Palo Alto, CA, USA) mRNA levels were used for normalization.

### Statistics

All experiments were repeated three times and were expressed as mean ± SD. *P* values were calculated using student's t test and *P* value<0.05 was considered significant. Statistical analysis was analyzed using the Statistical Package for Social Sciences (SPSS) software (version 16.0).

## Results

### Synergistic Antineoplastic Effects Induced by Decitabine and Gefitinib in Colon Cancer Cells

**Figure 1:**
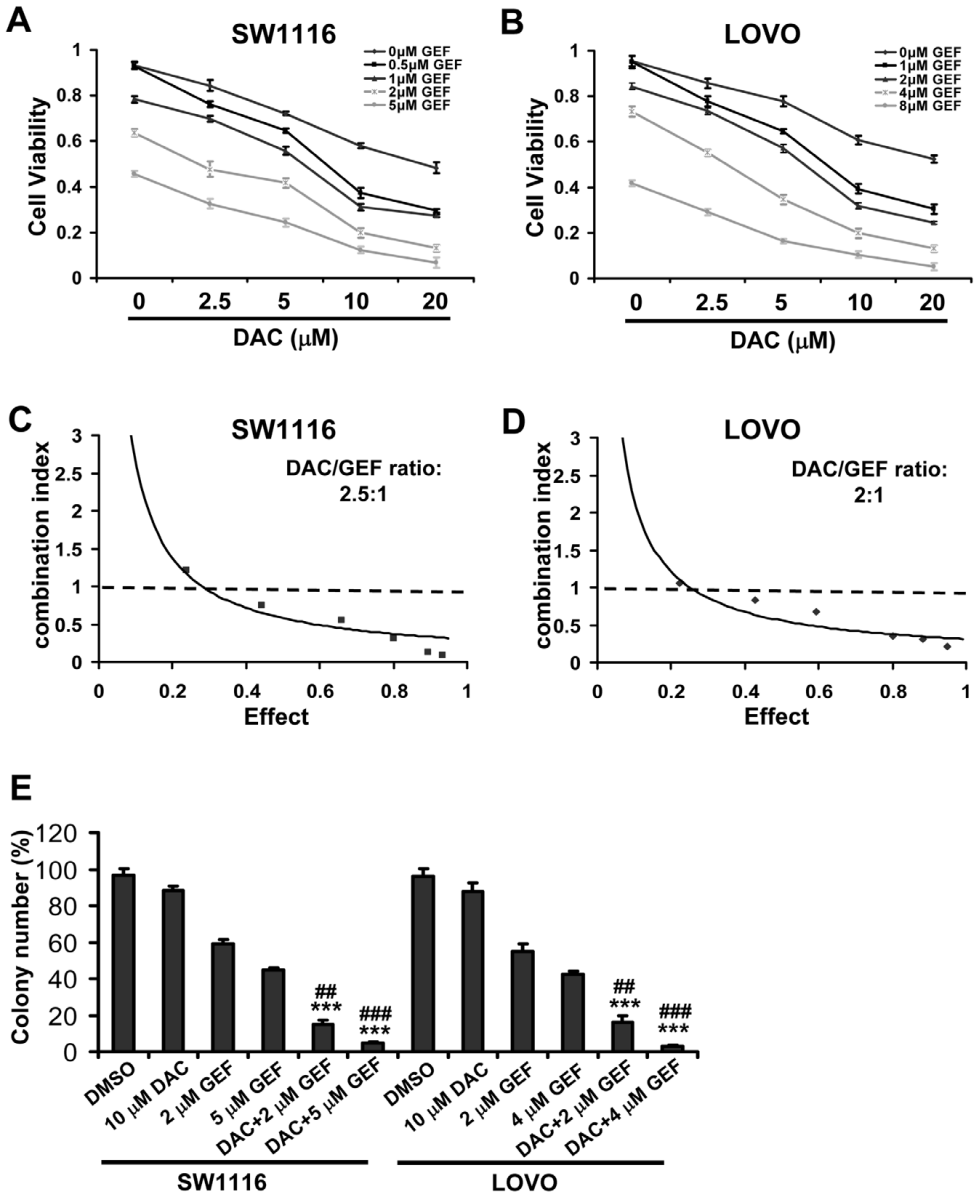
Synergistic antineoplastic effects of decitabine and gefitinib against colon cancer cells. (A) and (B) SW1116 and LOVO cells were cultured in control conditions (DMSO) or in the presence of the indicated concentrations of decitabine (DAC) and gefitinib (GEF), alone or in combination, for 48 h, and then assessed for viability by MTT assay. Results are means of duplicate assessments from one out of three independent experiments. (C) and (D) SW1116 cells and LOVO cells were plated, treated, and processed as in A and B. The dose–response curve of each drug was determined and combination index (CI) values for DAC/GEF concentration ratios (2.5∶1 in SW1116 cells, 2∶1 in LOVO cells) were calculated according to the Chou–Talalay's method at the 48 h time point, with the biological response being expressed as the fraction of affected cells. Rectangle symbol and diamond symbol designate the CI value for each fraction affected (effect). CI<1, CI = 1, CI>1 indicate synergistic, additive and antagonistic effects, respectively. The effect ranges from 0 (no inhibition) to 1 (complete inhibition). The data are representative of three independent experiments. (E) Influence of SW1116 cells and LOVO cells on the number of colony-forming cells, as evaluated by clonogenic assay. For colony-forming assay, the clonogenic assay was done as described in materials and methods. Columns, mean of three determinations; bars, SD. Results shown are representative of three independent experiments. **, *P*<0.01, ***, *P*<0.001, compared with DAC-treated cells. ##, *P*<0.01, ###, *P*<0.001, compared with GEF-treated cells.

To determine the effects of the combination treatment of DNA methyltransferase inhibitor decitabine and EGFR inhibitor gefitinib on human colon tumor cell viability, SW1116 [27] and LOVO cells [28] carrying wild-type *EGFR* gene were exposed to different concentrations of decitabine or gefitinib alone or in combination for up to 48 h, followed by the determination of cell viability using MTT assay. As shown in Fig. 1A and B, decitabine or gefitinib alone caused a concentration dependent inhibition of cell viability with IC_50_ values of 24.2 µM (decitabine) and 4.71 µM (gefitinib) in SW1116 cells, and IC_50_ values of 21.9 µM (decitabine) and 5.33 µM (gefitinib) in LOVO cells. Additionally, Fig. 1A and B showed that a combination of decitabine and gefitinib had a stronger inhibitory effect on the cell viability of SW1116 and LOVO cells than either compound alone. Furthermore, Fig. 1A indicated that treatment of SW1116 cells with fixed concentrations of decitabine decreased the IC_50_ values of gefitinib from 4.71 µM (in the absence of decitabine) to 1.25 µM (in the presence of 5 µM decitabine) and 0.19 µM (in the presence of 10 µM decitabine). Similarly, treatment of LOVO cells with fixed concentrations of decitabine decreased the IC_50_ values of gefitinib from 5.33 µM (in the absence of decitabine) to 0.63 µM (in the presence of 10 µM decitabine) and 0.12 µM (in the presence of 20 µM decitabine) (Fig. 1B).

These data suggested that the two compounds, decitabine and gefitinib, might synergize to inhibit cell viability in colon cancer cells. To confirm this synergism, we treated cells with a combination of the two agents in a constant ratio to one another and used Calcusyn software to calculate the combination index (CI) following Chou and Talalay's method as described under Methods. Fig. 1C and D revealed a significant synergy between the two agents (CI<1) in SW1116 and LOVO cells. In addition, by clonogenic cell survival assay, we found that decitabine and gefitinib exerted synergistic effects to inhibit the clonogenic activity of SW1116 and LOVO cells (Fig. 1E). Furthermore, the few cells surviving decitabine plus gefitinib generated colonies that were much smaller in size than those generated by cells surviving either of these agents alone (data not shown). Notably, when used together, treatment of NCM460 cells, a normal human colon mucosal epithelial cell line, with decitabine and gefitinib showed an effect greater than when each compound was used individually, but effects were less than additive suggesting antagonism (Fig. S1A and B). Moreover, the combination of low concentration of decitabine (2.5 µM) and gefitinib (1 µM for LOVO cells and 0.5 µM for SW1116 cells) efficiently abrogated cell migration in a synergic manner (Fig. S2A). Meanwhile, we detected the cell viability of colon cancer cells treated using the two agents alone or in combination (Fig. S2B), and found that the combination of low concentration of decitabine and gefitinib did not significantly decrease cell viability. These results indicated that the reduction of cells migration caused by the two drugs was not involved in inhibition of cell viability.

### Decitabine and Gefitinib Combination Treatment is More Effective at Inhibiting AKT and mTOR Signaling Pathways in Colon Cancer Cells

**Figure 2:**
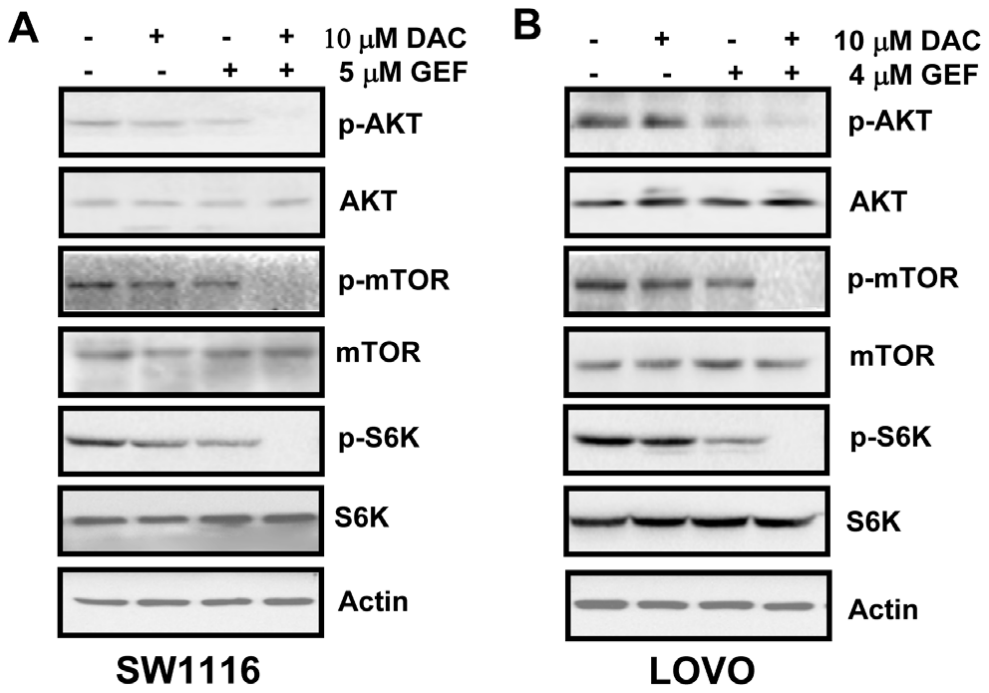
The combination of decitabine and gefitinib inhibits AKT and mTOR signaling pathways. (A) and (B) SW1116 and LOVO cells were plated, treated for 48 h with decitabine (DAC) and gefitinib (GEF) either alone or in combination, and the expression levels of AKT, mTOR, S6K, and phosphorylation were determined by Western blot analysis as described under Methods. Expression of β-actin served as a loading control. The data are representative of three independent experiments.

Decitabine and gefitinib significantly inhibited the growth of two types of colon cancer cells compared to the treatment with either agent alone. As AKT and mTOR signaling pathways play a critical role in cell growth and cell apoptosis, we determined the effects of decitabine and gefitinib on the activation of these pathways. We calculated CI values to further find that the combination of 10 µM decitabine with 5 µM gefitinib in SW1116 cells or 4 µM gefitinib in LOVO cells was the most effective. SW1116 and LOVO cells were treated with decitabine and gefitinib either alone or in combination. After 48 h, the cells were processed for Western blot as described under methods. As shown in Fig. 2A and B, decitabine (10 µM) could not lead to significant alterations in AKT, or mTOR activity, as assessed by Western blot for phosphorylation of AKT, mTOR and S6K. Additionally, we observed minimal reductions of phosphorylation of AKT, mTOR and S6K with 5 µM gefitinib in SW1116 and 4 µM in LOVO cells. However, the combination of the two drugs completely abrogated AKT and mTOR activities in SW1116 and LOVO cells (Fig. 2A and B).

### Decitabine Synergistically Enhances Gefitinib-induced Apoptosis in Colon Cancer Cells

**Figure 3:**
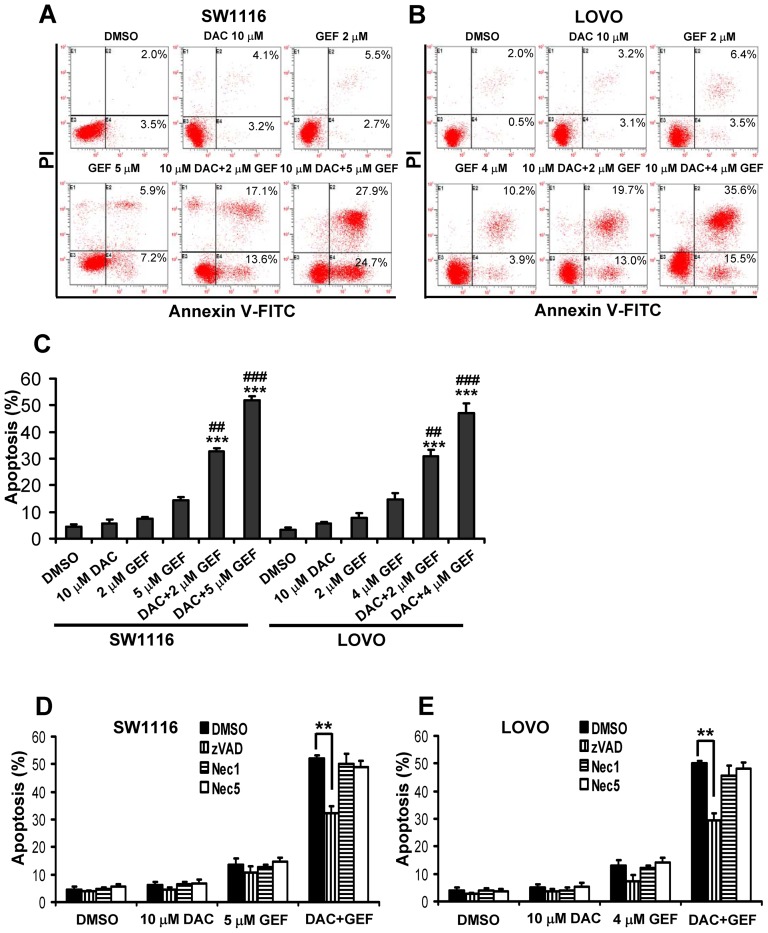
Decitabine synergistically enhances gefitinib-induced apoptosis in colon cancer cells. (A) and (B) SW1116 and LOVO cells were cultured in control conditions (DMSO) or in the presence of the indicated concentrations of decitabine (DAC) and gefitinib (GEF), alone or in combination, for 48 h. And then cells were stained with Annexin V-FITC and propidium iodide (PI) and analyzed by flow cytometry. This experiment was done in triplicate and representative diagrams of Annexin V-FITC assays are shown. (C) Quantitative measurement of Annexin V-FITC flow cytometry analyses showed positive apoptotic cells in response to DAC and GEF, alone or in combination. Columns, mean; bars, SD. **, *P*<0.01, ***, *P*<0.001, compared with DAC-treated cells. ##, *P*<0.01, ###, *P*<0.001, compared with GEF-treated cells. (D) and (E) SW1116 and LOVO cells were pre-treated with 10 µM z-VAD-fmk (zVAD) or 20 µM necrostatin-1 (Nec1) or necrostatin-5 (Nec5) for 1 h followed by treatment with the indicated concentrations of DAC and GEF, alone or in combination, for additional 48 h. And then the apoptotic cells were determined by Annexin V-FITC/PI staining and flow cytometry analysis. This experiment was repeated thrice. Columns, mean; bars, SD. **, *P*<0.01.

To determine if the cytotoxic effects of gefitinib combined with decitabine were due to induction of apoptosis, SW1116 and LOVO cells were treated with the two compounds, alone or in combination, for 48 h and then cell apoptosis was determined by Annexin V-FITC and propidium iodide (PI) staining and flow cytometry analysis. As shown in Fig. 3A and C, treatment with gefitinib (2 µM or 5 µM) or decitabine (10 µM) alone had weak effects on apoptosis in SW1116 cells. However, there was a significantly higher apoptosis rate found upon treatment with the combination of gefitinib and decitabine (Fig. 3A and C). Similar results were found in LOVO cells (Fig. 3B and C). Additionally, cell apoptosis was measured by detecting sub-G1 population with PI staining and flow cytometry analyses. As shown in Fig. S3, the sub-G1 population percentages induced by the treatments with the two drugs combination were greater than those induced by the drugs individually.

To determine whether or not gefitinib plus decitabine caused caspase cascade, the pan caspase inhibitor z-VAD-fmk (10 µM) was used to pretreat SW1116 or LOVO cells before treatment of gefitinib plus decitabine. As shown in Fig. 3D and E, z-VAD-fmk could remarkably restrain cell apoptosis induced by gefitinib plus decitabine. However, the cell apoptosis triggered by the two compounds in combination could not be blocked by necrostatin-1 or necrostatin-5, a novel class of potent small-molecule inhibitors of cell necrosis. These results revealed that the apoptotic pathway was involved in the cell death induced by gefitinib combined with decitabine, in colon cancer cells.

### Decitabine and Gefitinib Combination Therapy Alters the Expression Levels of Apoptotic Regulatory Factors in Colon Cancer Cells

**Figure 4:**
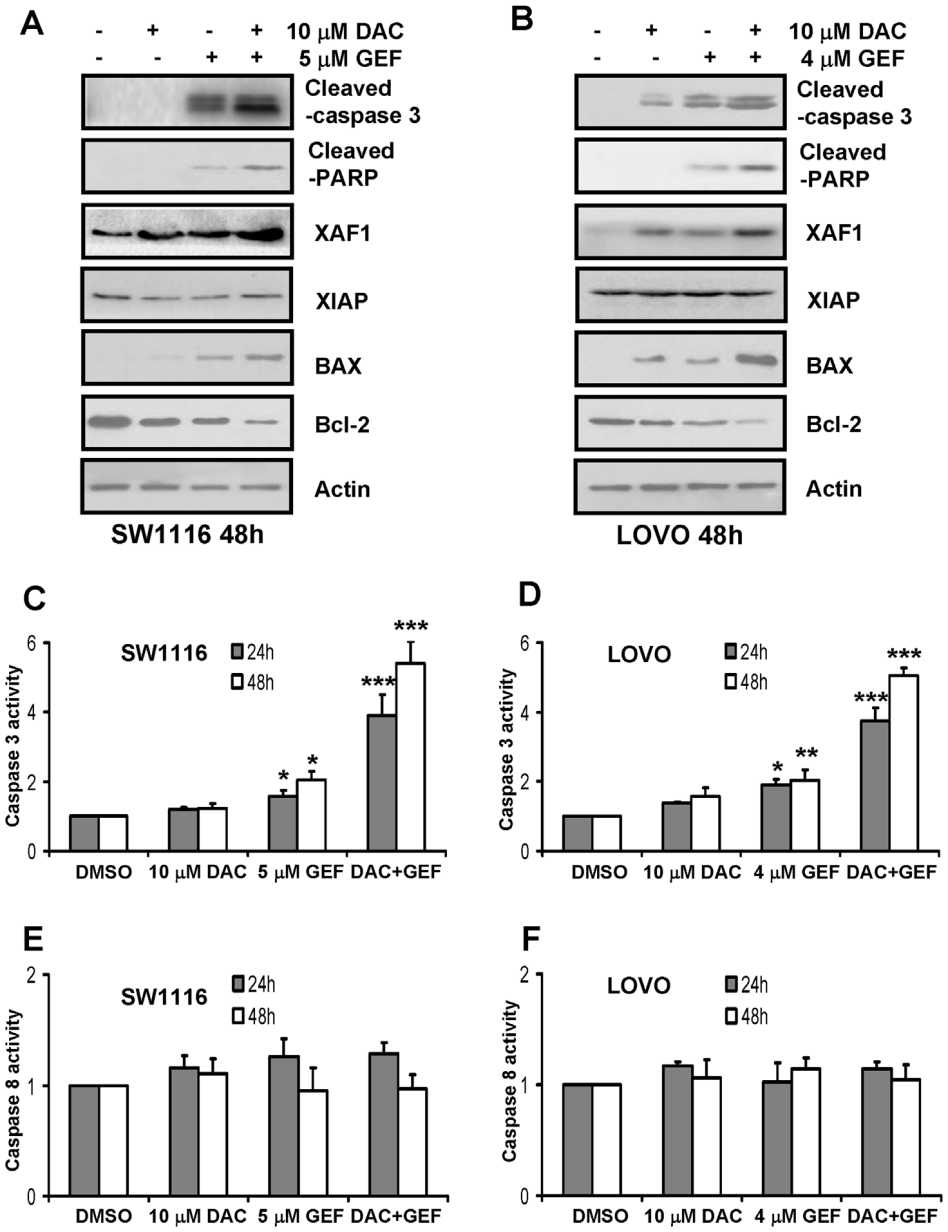
Decitabine and gefitinib combination therapy alters the expression levels of apoptotic regulatory factors. SW1116 and LOVO cells were plated, treated for 48(DAC) and gefitinib (GEF) either alone or in combination. (A) and (B) the expression levels of cleaved-caspase 3, cleaved-PARP, XAF1, XIAP, BAX, Bcl-2 were determined by Western blot analysis as described under Methods. Expression of β-actin served as a loading control. The data are representative of three independent experiments. (C) and (D) The caspase 3 activity was quantified as described under methods. This experiment was repeated thrice. Columns, mean; bars, SD. *, *P*<0.05; **, *P*<0.01; ***, *P*<0.001. (E) and (F) The caspase 8 activity was quantified as described under Methods. This experiment was repeated thrice. Columns, mean; bars, SD. *, *P*<0.05; **, *P*<0.01; ***, *P*<0.001.

Since apoptosis is tightly regulated by pro- and antiapoptotic members of the Bcl-2 protein family, the proapoptotic factors BAX, BID and BIM as well as the antiapoptotic proteins Bcl-2 and Bcl-XL were studied in SW1116 and LOVO cells after treatment with decitabine or gefitinib or their combination by Western blot analysis. SW1116 and LOVO cells responding to decitabine plus gefitinib manifested increased amounts of the proapoptotic BAX as well as major reduction in the levels of the anti-apoptotic protein Bcl-2 (Fig. 4A and B). However, the two compounds in combination failed to affect the expression levels of other members of the Bcl-2 protein family, including the proapoptotic proteins BID and BIM as well as antiapoptotic protein Bcl-XL (data not shown).

Inhibitor of apoptosis protein (IAP) is a protein family acting through the inhibition of caspase activity. The X-linked IAP (XIAP), a member of IAP, and XIAP-associated factor 1 (XAF1) were examined in colon cancer cells stimulated with decitabine along with gefitinib. Fig. 4A and B indicated that no changes in XIAP protein levels were found in SW1116 and LOVO cells treated by decitabine and gefitinib alone or in combination. Notably, combined treatment with both drugs remarkably increased the expression of XAF1 compared to single agent treatment (Fig. 4A and B).

To test the ability of gefitinib combined with decitabine to activate caspases, cleaved caspase 3 and cleaved PARP were studied in SW1116 and LOVO cells after treatment with gefitinib or decitabine or their combination by Western blot analysis. Significant increase in the amounts of both cleaved caspase 3 and cleaved PARP were noted in colon cancer cells treated with two drugs in combination compared to treatment with single agents (Fig. 4A and B). Moreover, colon cancer cells treated by the two compounds were analysed for caspase 3 and caspase 8 activities by fluorogenic substrate cleavage. As shown in Fig. 4C and D, colon cancer cells treated with two compounds in combination showed significant increase in caspase 3 activity. Additionally, decitabine plus gefitinib exerted time-dependent caspase 3 activity inductions on SW1116 and LOVO cells (Fig. 4C and D). In contrast, no changes were found in caspase 8 activities (Fig. 4E and F).

### XAF1 Plays a Critical Role in Cellular Apoptosis Triggered by Decitabine Combined with Gefitinib

**Figure 5:**
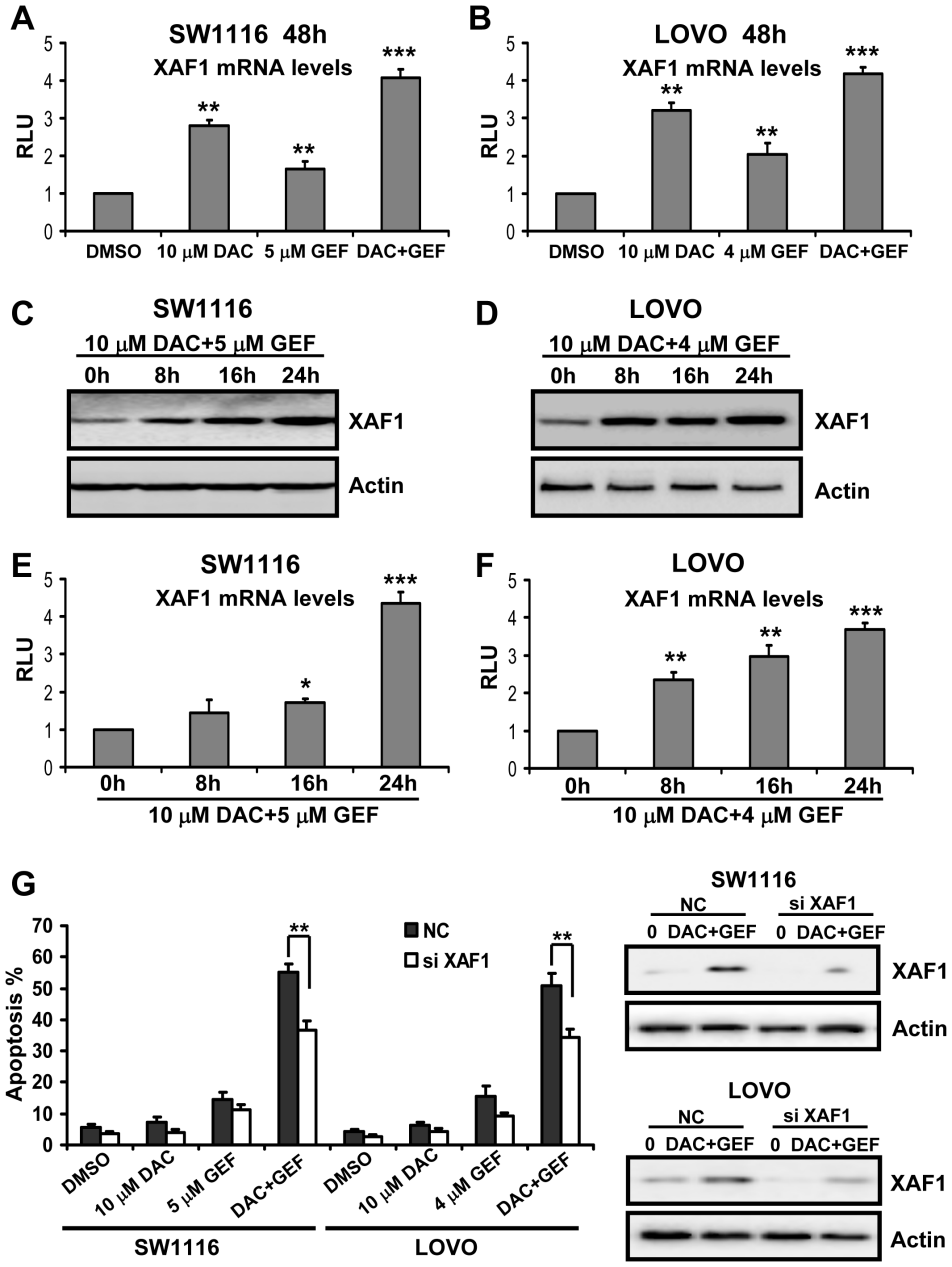
XAF1 plays a critical role in cellular apoptosis triggered by decitabine combined with gefitinib. (A) and (B) SW1116 and LOVO cells were treated for 48 h with the indicated concentrations of decitabine (DAC) and gefitinib (GEF) either alone or in combination. The mRNA expression levels of XAF1 were determined by real-time quantitative PCR. Expression of β-actin served as control. The data are representative of three independent experiments. Columns, mean; bars, SD. **, *P*<0.01; **, *P*<0.001. (C) and (D) SW1116 and LOVO cells were treated for the indicated time intervals with the indicated concentrations of DAC and GEF in combination. The expression levels of XAF1 were determined by Western blot analysis as described under methods. Expression of β-actin served as a loading control. The data are representative of three independent experiments. (E) and (F) SW1116 and LOVO cells were treated for the indicated time intervals with the indicated concentrations of DAC and GEF in combination. The mRNA expression levels of XAF1 were determined by real-time quantitative PCR. Expression of β-actin served as control. The data are representative of three independent experiments. Columns, mean; bars, SD. *, *P*<0.05; **, *P*<0.01; ***, *P*<0.001. (G) SW1116 and LOVO cells were treated with 100 nM XAF1 siRNA and negative control (NC) siRNA for 12 h, and treated with the indicated concentrations of DAC and GEF in combination for an additional 48 h. And then the apoptotic cells were determined by Annexin V-FITC/PI staining and flow cytometry analysis (left panel). This experiment was repeated thrice. Columns, mean; bars, SD. **, *P*<0.01. The knockdown effects on XAF1 were confirmed by Western blot analysis (right panel). The data are representative of three independent experiments.

The data shown above indicated that decitabine combined with gefitinib increased the XAF1 levels in SW1116 and LOVO cells. We then asked if the two drugs in combination could enhance XAF1 mRNA levels in colon cancer cells. As shown in Fig. 5A and B, XAF1 mRNA levels were increased significantly by decitabine plus gefitinib. Furthermore, colon cancer cells were treated with the two drugs in combination for different time intervals. We showed that XAF1 protein and mRNA levels were increased by decitabine plus gefitinib in a time-dependent manner (Fig. 5C, D, E and F).

To more directly assess the role of XAF1 in the apoptotic activity of the decitabine combined with gefitinib, cell apoptosis was evaluated in colon cancer cells treated with the two drugs in combination with siRNA-mediated knockdown of XAF1. As shown in Fig. 5G, knockdown of XAF1 significantly attenuated cell apoptosis induced by gefitinib treatment in combination with decitabine in colon cancer cells. Together, these findings suggested that XAF1 contributed to the sensitivity of colon cancer cells to apoptotic induction after combined treatment with gefitinib and decitabine.

## Discussion

There have been multiple investigations of chemotherapeutics that target EGFR and thereby attenuating the EGFR signaling pathway in colon cancer [29]. However, response effects for single EGFR inhibitor remain relatively modest unless the EGFR-targeted therapy is combined with other chemotherapeutics [30], [31],[32],[33]. Consistent with previous findings, we found that EGFR inhibitor gefitinib alone modestly decreased the cell viability in SW1116 and PC-9 colon cancer cell lines carrying wild-type *EGFR* gene [27], [28]. DNA methyltransferase inhibitors as promising anti-tumor agents have been shown to demethylate and upregulate the expression of tumor suppressor genes and display strong anti-tumor growth effect *in vitro*, *in vivo* and in the clinic [34]. Although DNA demethylating agents induced apoptosis are minimal on its own, they exert potential to enhance the effects of other chemotherapeutics, such as DNA damaging agent cisplatin [35].

In the current study, decitabine combined with gefitinib was more effective to inhibit cell viability than single agent alone in colon cancer cells. Combination index data analysis showed that this combination is highly synergistic at inhibiting cell viability of colon cancer cells. Clonogenic assay results showed the number of positive colonies was strikingly reduced in the cells treated with the combination therapy, suggesting that the damage inflicted by the interaction of treatments was chronic and that the affected cells were not able to recover. Furthermore, Annexin V assay showed that the combination of decitabine plus gefitinib was synergistic at inducing apoptosis in colon cancer cells. These results raised the possibility that the combination of decitabine and gefitinib exerted a dual anticancer action in colon cancer cells, which consisted in cell apoptosis induction plus cell-cycle blockade. Additionally, the combination was synergistic at inhibiting colon cancer cells migration. More importantly, the combination of decitabine and gefitinib displayed minimal toxicity to NCM460 cells, a normal human colon mucosal epithelial cell line. The data shown above indicated a novel therapeutic strategy using a combination of decitabine and gefitinib in human colon cancer.

Our results showed that gefitinib inhibited the expression of p-AKT, p-mTOR and p-S6K, main mediators of AKT/mTOR pathway. Moreover, combined treatment with decitabine and gefitinib decreased the expression of p-AKT, p-mTOR and p-S6K more than single agent alone, indicating synergistic suppression of AKT/mTOR pathway. It has been shown that AKT/mTOR pathway is critical for cell survival and resistance to apoptosis [36], [37], [38]. Thus, cells apoptosis and inhibition of cells survival induced by two agents in combination could be involved in inhibiting the AKT/mTOR pathway. AKT has been shown to directly interact with and phosphorylate XIAP [39], [40]. XIAP phosphorylated by AKT can prevent XIAP degradation and thus inhibit caspase 3 activation confer resistance to apoptosis [41]. Therefore, we examined the expression of XIAP in colon cancer cells treated by the two agents alone or in combination. Unexpectedly, we did not detect an obvious reduction of XIAP expression in the colon cancer cells treated by two compounds in combination. One possible explanation for the discrepancy of our results and previous studies might be due to the interaction between XIAP and AKT was in specific histologic types of cancer or in cell line specific pathways.

Several studies indicated that cell cycle regulatory gene *CDKN2A* is hypermethylated in colon cancer cells [42], and decitabine-induced cell proliferation inhibition may result from the release of methylation silencing of the *CDKN2A* gene [43]. In addition, previous studies suggested that allelic loss of the *XAF1* gene is prevalent in cancer cell lines [44]. In contrast, XIAP levels are relatively high in the majority of cancer cell lines [45]. Studies suggested that a high level of XIAP to XAF1 expression in cancer cells may provide a survival advantage through the relative increase of XIAP anti-apoptotic function [46], [47]. Moreover, in many colon cancers, the CpG island of *XAF1* gene is hypermethylated, resulting in transcriptional repression [48], [49], [50]. In the present study, the expression of XAF1 was induced by DNA demethylating agent decitabine in SW1116 and LOVO cells, raising a possibility that decitabine increased XAF1 level by preventing the CpG island methylation of *XAF1* gene. More importantly, the expression of both mRNA and protein levels of XAF1 was remarkably increased in colon cancer cells treated by using two drugs in combination compared to single agent treatment. Gefitinib is known to inhibit transmembrane transporters of the ABC family, including the P-gp, MRP1 and BCRP [51]. This raised a possibility that gefitinib incremented the cellular accumulation of decitabine correlating with its capacity to inhibit cell proliferation by enhancing the CDKN2A expression and induce cell apoptosis by upregulating the XAF1 level.

Notably, the expression of XAF1 was upregulated modestly by gefitinib alone in colon cancer cells, raising another likely explanation for the upregulation of XAF1 was that gefitinib also targeted epigenetic pathways. Further investigation is needed to study the mechanism by which XAF1 was induced by decitabine and gefitinib alone or in combination. To ask whether the induction of apoptosis by decitabine in combination with gefitinib was involved in XAF1, XAF1 was depleted by using siRNA in colon cancer cells. We found that depletion of XAF1 by siRNA attenuated apoptosis of colon cancer cell induced by decitabine in combination with gefitinib. These results suggested XAF1 contributed to the cell apoptosis triggered by the two agents in combination.

Annexin-V assay revealed SW1116 and LOVO cells apoptosis induced by combination treatment of two agents. In addition, decitabine plus gefitinib exerted apoptotic effects, as indicated by the fact that the broad-spectrum caspase inhibitor z-VAD-fmk could prevent cells death as induced by these agents. However, cells death induced by decitabine combined with gefitinib failed to be blocked by necrostatin-1 or necrostatin-5, a novel class of potent small-molecule inhibitors of necroptosis [52], [53]. To further address the underlying mechanism of this enhanced cell apoptosis, the expression and processing of caspase 3 and PARP were examined in colon cancer cells. Both caspase 3 and PARP are cleaved in the late “execution” apoptotic phase and the combination of decitabine and gefitinib synergistically induced cleavage of caspase 3 and PARP. Moreover, decitabine also synergistically enhanced the ability of gefitinib to increase caspase 3 activity. However, caspase 8 activity could not be induced by these agents. Additionally, we found that the combination of decitabine and gefitinib could lead to marked inhibition of Bcl-2 and upregulation of BAX, both of which would serve to facilitate apoptotic induction. Overall, our study showed that the combination of decitabine and gefitinib synergistically induced cell apoptosis was involved in mitochondria-mediated apoptotic pathway in colon cancer cells.

In this study, we demonstrated the synergistic effects of decitabine and gefitinib in inhibiting cell growth, inducing cell apoptosis, and suppressing cell migration in colon cancer cells. Decitabine in combination with gefitinib inhibited cell growth and induced cell apoptosis partly through inhibition of the AKT/mTOR pathway. Moreover, our study results suggested that mitochondria-mediated and XAF1-dependent apoptotic pathways were involved in decitabine plus gefitinib-induced cell death in colon cancer cells. In summary, this study provided preclinical evidence that the combination of decitabine and gefitinib could be a novel and promising therapeutic approach to the treatment of colon cancer, which warrants further investigation in a clinical setting.

## Supporting Information

Figure S1 **Anti-proliferation effects of decitabine and gefitinib against NCM460 cells.** (A) NCM460 cells were cultured in control conditions (DMSO) or in the presence of the indicated concentrations of decitabine (DAC) and gefitinib (GEF), alone or in combination, for 48 h, and then assessed for viability by MTT assay. Results are means of duplicate assessments from one out of three independent experiments. (B) NCM460 cells were plated, treated, and processed as in A. The dose–response curve of each drug was determined and combination index (CI) values for DAC/GEF concentration ratios (2.5∶1) were calculated according to the Chou–Talalay's method at the 48 h time point, with the biological response being expressed as the fraction of affected cells. Rectangle symbol designates the CI value for each fraction affected (effect). CI<1, CI = 1, CI>1 indicate synergistic, additive and antagonistic effects, respectively. The effect ranges from 0 (no inhibition) to 1 (complete inhibition). The data are representative of three independent experiments.
(TIF)

Figure S2 **Decitabine synergistically enhances gefitinib-inhibited cell migration in colon cancer cells.** (A) and (B) SW1116 and LOVO cells were treated with the indicated concentrations of decitabine (DAC) and gefitinib (GEF) either alone or in combination for 24 h. The migratory properties of cells were analyzed by transwell assay. Data summarized three independent experiments. (C) and (D) SW1116 and LOVO cells were treated with the indicated concentrations of DAC and GEF either alone or in combination for 24 h. Proliferation of SW1116 and LOVO cells is shown. Average of three independent experiments is shown.
(TIF)

Figure S3 **Decitabine synergistically enhances gefitinib-induced apoptosis in colon cancer cells.** SW1116 and LOVO cells were treated with the indicated concentrations of decitabine (DAC) and gefitinib (GEF) either alone or in combination for 48 h. Apoptosis was measured by detecting sub-G1 population with propidium iodide (PI) staining and flow cytometry analyses as described in materials and methods. Columns, mean of three determinations; bars, SD. Results shown are representative of three independent experiments. ***, *P*<0.001, compared with DAC-treated cells. ##, *P*<0.01, ###, *P*<0.001, compared with GEF-treated cells.
(TIF)

Materials and Methods S1 (DOC)
