# Supplementary figures and images for: Combination of Gefitinib and DNA Methylation Inhibitor Decitabine Exerts Synergistic Anti-Cancer Activity in Colon Cancer Cells
Source: PLoS One. 2014 May 29;9(5):e97719. doi: 10.1371/journal.pone.0097719 (PMC4038521; doi:10.1371/journal.pone.0097719)

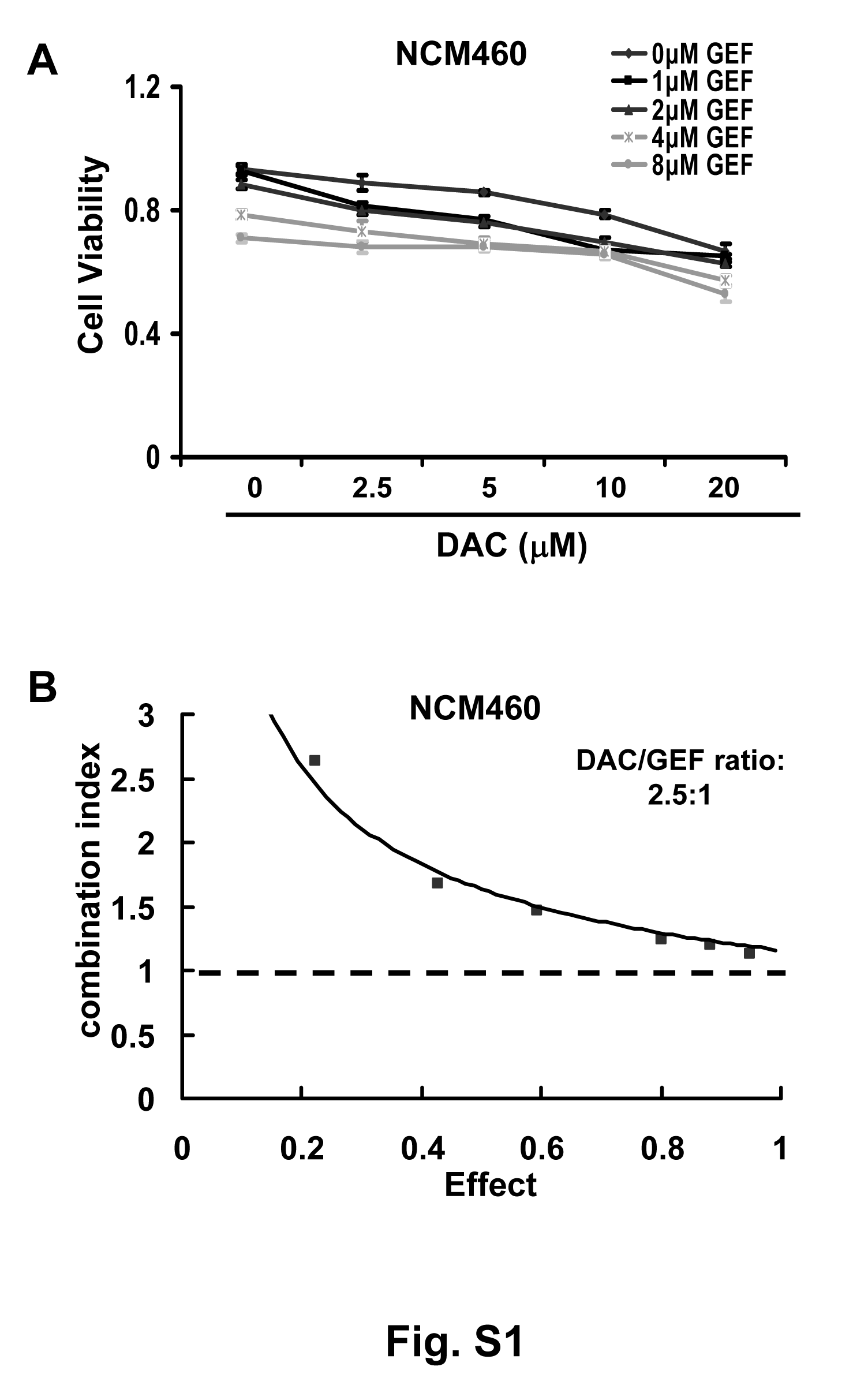

Supplement: Figure S1 — Anti-proliferation effects of decitabine and gefitinib against NCM460 cells. (A) NCM460 cells were cultured in control conditions (DMSO) or in the presence of the indicated concentrations of decitabine (DAC) and gefitinib (GEF), alone or in combination, for 48 h, and then assessed for viability by MTT assay. Results are means of duplicate assessments from one out of three independent experiments. (B) NCM460 cells were plated, treated, and processed as in A. The dose–response curve of each drug was determined and combination index (CI) values for DAC/GEF concentration ratios (2.5∶1) were calculated according to the Chou–Talalay's method at the 48 h time point, with the biological response being expressed as the fraction of affected cells. Rectangle symbol designates the CI value for each fraction affected (effect). CI<1, CI = 1, CI>1 indicate synergistic, additive and antagonistic effects, respectively. The effect ranges from 0 (no inhibition) to 1 (complete inhibition). The data are representative of three independent experiments. (TIF) [file pone.0097719.s001.tif]

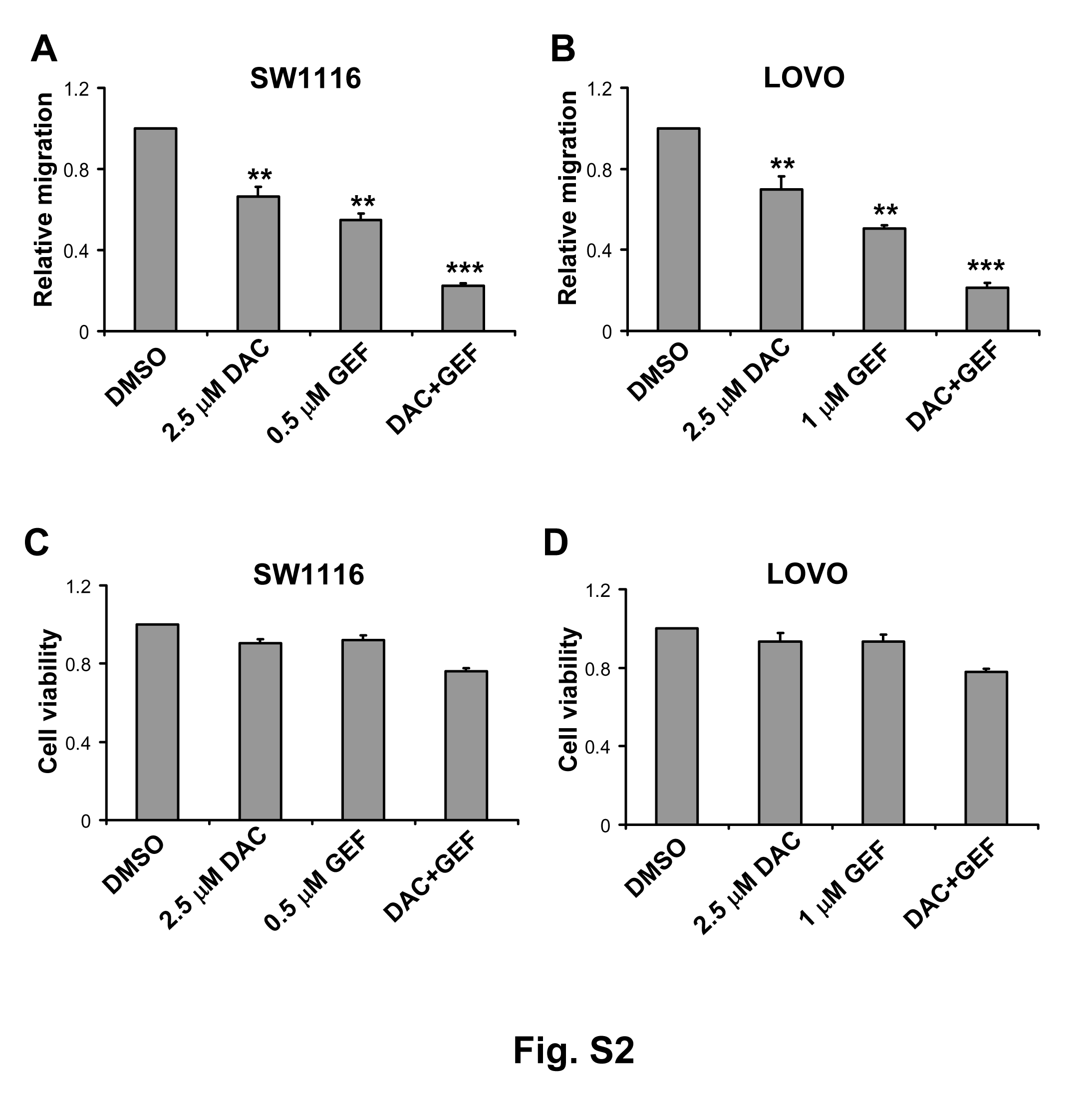

Supplement: Figure S2 — Decitabine synergistically enhances gefitinib-inhibited cell migration in colon cancer cells. (A) and (B) SW1116 and LOVO cells were treated with the indicated concentrations of decitabine (DAC) and gefitinib (GEF) either alone or in combination for 24 h. The migratory properties of cells were analyzed by transwell assay. Data summarized three independent experiments. (C) and (D) SW1116 and LOVO cells were treated with the indicated concentrations of DAC and GEF either alone or in combination for 24 h. Proliferation of SW1116 and LOVO cells is shown. Average of three independent experiments is shown. (TIF) [file pone.0097719.s002.tif]

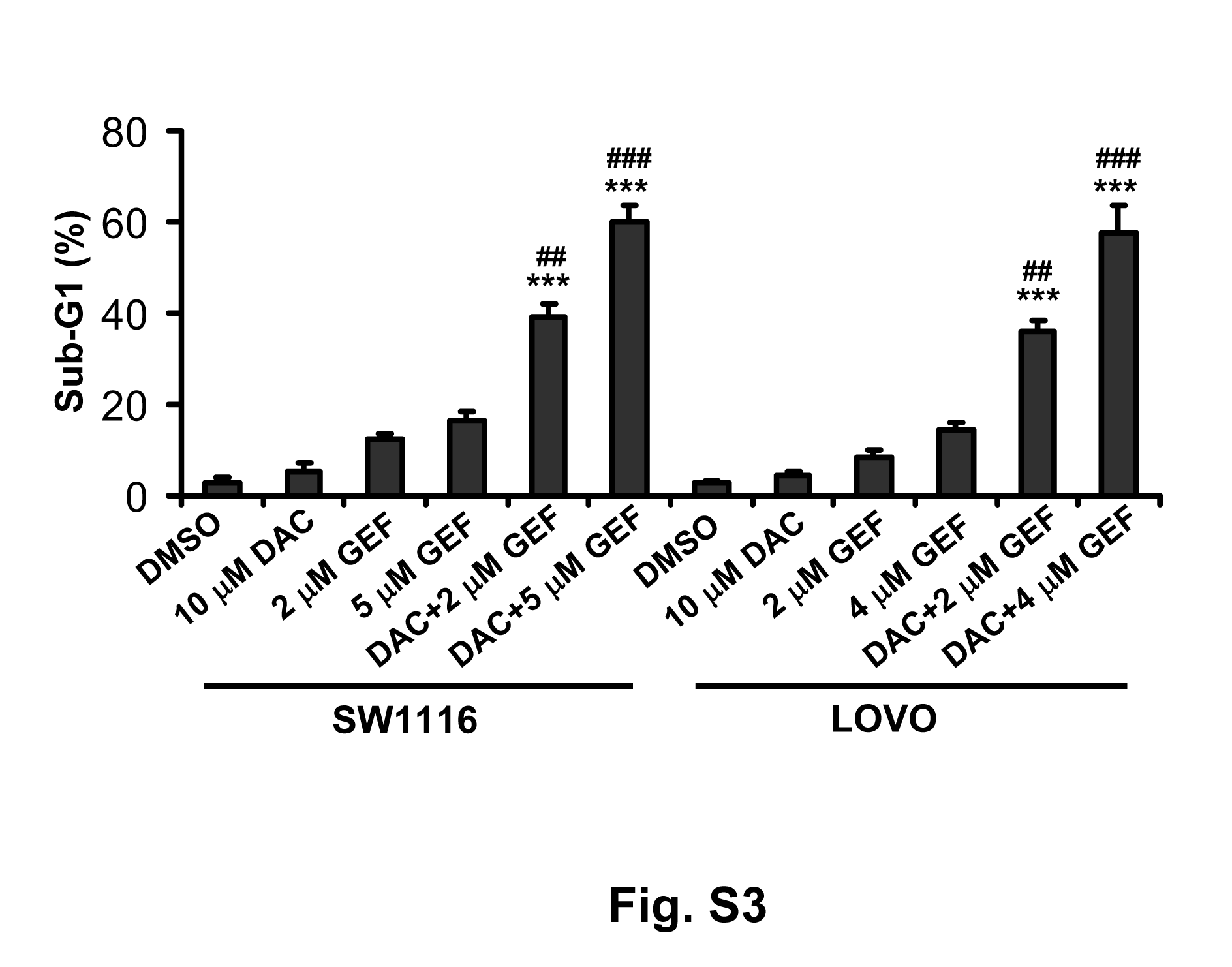

Supplement: Figure S3 — Decitabine synergistically enhances gefitinib-induced apoptosis in colon cancer cells. SW1116 and LOVO cells were treated with the indicated concentrations of decitabine (DAC) and gefitinib (GEF) either alone or in combination for 48 h. Apoptosis was measured by detecting sub-G1 population with propidium iodide (PI) staining and flow cytometry analyses as described in materials and methods. Columns, mean of three determinations; bars, SD. Results shown are representative of three independent experiments. ***, P<0.001, compared with DAC-treated cells. ##, P<0.01, ###, P<0.001, compared with GEF-treated cells. (TIF) [file pone.0097719.s003.tif]
