# Supplementary material for: Combination of Gefitinib and DNA Methylation Inhibitor Decitabine Exerts Synergistic Anti-Cancer Activity in Colon Cancer Cells
Source: PLoS One. 2014 May 29;9(5):e97719. doi: 10.1371/journal.pone.0097719 (PMC4038521; doi:10.1371/journal.pone.0097719)
Supplement: Materials and Methods S1 — (DOC) [file pone.0097719.s004.doc]

**Materials and Methods**

**Transwell Migration Assay**

Transwell assay was performed as described previously [1]. Briefly, cells were incubated in serum or serum-free media containing desired drugs for 16 h. The migrated cells in five fields were counted, and the average of each chamber was determined.

**Sub-G1 Population Assay**

Sub-G1 population was analyzed by flow cytometer. Every well of a 6-well plate was seeded with 5×10^5^/mL cells. Then cells were treated with the indicated concentration of drugs. After 48 h, cells were harvested and fixed using 70% ethanol at -20 °C for 24 h. And then cells were stained with mixture of 50 μg/mL propidium iodide, 0.2% Triton X-100, and 1 mg/mL RNAase, and performed using a FACS flow cytometer equipped with Modfit LT for Mac V2.0 software (BD Biosciences, San Jose, CA).

**References:**

1. Yang KL, Wang YS, Chang CC, Huang SC, Huang YC, et al. (2013) Reciprocal complementation of the tumoricidal effects of radiation and natural killer cells. PLoS One 8: e61797.
